# Icaritin Reduces Oral Squamous Cell Carcinoma Progression via the Inhibition of STAT3 Signaling

**DOI:** 10.3390/ijms18010132

**Published:** 2017-01-12

**Authors:** Jian-Guang Yang, Rui Lu, Xiao-Jing Ye, Jing Zhang, Ya-Qin Tan, Gang Zhou

**Affiliations:** 1 The State Key Laboratory Breeding Base of Basic Science of Stomatology (Hubei-MOST) and Key Laboratory of Oral Biomedicine Ministry of Education, School and Hospital of Stomatology, Wuhan University, Wuhan 430079, China; 2014103040017@whu.edu.cn (J.-G.Y.); KQ000874@whu.edu.cn (R.L.); 2014103040018@whu.edu.cn (X.-J.Y.); zhang_lin_ling@whu.edu.cn (J.Z.); 2015103040008@whu.edu.cn (Y.-Q.T.); 2 Department of Oral Medicine, School and Hospital of Stomatology, Wuhan University, Wuhan 430079, China

**Keywords:** icaritin, oral squamous cell carcinoma, signal transducer and activator of transcription 3, premalignant lesion

## Abstract

Icaritin, a traditional Chinese medicine, possesses antitumor activity. The current study aimed to investigate icaritin effect and potential mechanism on oral squamous cell carcinoma (OSCC) development. OSCC cells proliferation, apoptosis, and autophagy were analyzed after incubation with icaritin at different concentrations and incubation times. The expressions of proteins related to proliferation, apoptosis, and autophagy, as well as signal transducer and activator of transcription 3 (STAT3) signal network, were also evaluated by western blot. Furthermore, STAT3 was knocked down by siRNA transfection to determine STAT3 role in OSCC cell proliferation and apoptosis. An oral specific carcinogenesis mouse model was used to explore icaritin effect on OSCC in vivo. Icaritin significantly inhibited OSCC proliferation in vitro and reduced the expression of both the cell-cycle progression proteins cyclin A2 and cyclin D1. Besides, icaritin increased cleaved caspase 3 and cleaved poly-(ADP-ribose) polymerase expression leading to apoptosis, and it activated autophagy. Icaritin significantly inhibited the expression of phospho-STAT3 (p-STAT3) in a dose- and time-dependent manner. In the in vivo experiment, the number of malignant tumors in the icaritin-treated group was significantly lower than the control. Overall, icaritin suppressed proliferation, promoted apoptosis and autophagy, and inhibited STAT3 signaling in OSCC in vitro and in vivo. In conclusion, icaritin might be a potential therapeutic agent against OSCC development.

## 1. Introduction

Oral squamous cell carcinoma (OSCC), as one of the most common cancers in the world, is a major public health problem [1]. OSCC worldwide incidence exceeds 300,000 cases and about 145,000 of them die annually [2]. Despite recent therapeutic advances in oral cancer, the mortality and over five-year survival rates remain unchanged, which can be largely attributed to the local recurrences and distant metastasis [3,4]. During the multistep carcinogenesis, the oral cavity exhibits clinically defined premalignant lesions such as oral leukoplakia and erythroplakia [5]. The premalignant lesions are believed to have high risk associated with the progression to cancer [6]. Thus, targeting these lesions may represent a good option to prevent tumor progression and ultimately reduce the incidence of OSCC. Therefore, new and effective therapeutic agents guaranteeing safety and minimal toxicity are urgently required for OSCC prevention and treatment.

**Figure 1:**
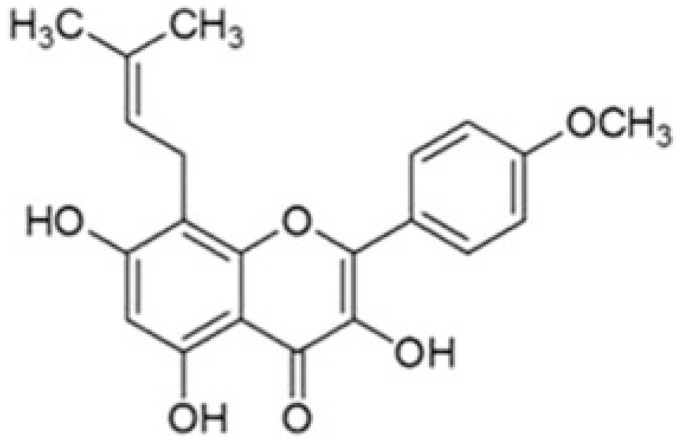
Chemical structure of icaritin.

Icaritin is a hydrolytic product of icarin from Epimedium Genus, a traditional Chinese herbal medicine [7] (Figure 1). Previous studies showed that icaritin has various pharmacological and biological effects, including cardiovascular function improvement, hormone regulation, and modulation of immunological function [8]. In addition, it induces apoptosis and growth inhibition, downregulates tumor angiogenesis, and inhibits invasion and epithelial-to-mesenchymal transition in human cancer cells, such as prostate carcinoma cells, endometrial cancer cells, breast cancer stem/progenitor cells, hematological malignancy cells, renal cell carcinoma cells, lung cancer cells, and multiple myeloma cells [9,10,11,12,13,14,15,16,17].

Recent studies revealed that signal transducer and activator of transcription 3 (STAT3) is constitutively activated and involved in the process of apoptosis resistance in many malignant tumors [18,19,20,21]. STAT3 is also well-known for its role in cancer-related inflammation, immunity, and autophagy [22,23]. Janus kinases JAKs phosphorylate gp130, leading to the recruitment and activation of STAT3, which then result in STAT3-mediated transcriptional regulation [18,22]. Some reports suggested that OSCC tumor cells frequently produce high levels of p-STAT3 [18,20], and various JAKs and STAT3 inhibitors could abrogate OSCC growth [19,24,25,26,27]. In addition, icaritin exerted an anti-tumor effect via inhibiting JAKs/STAT3 activation [12,14,16]. Thus, on the basis of this evidence, we hypothesized that the inhibition of JAK2/STAT3 aberrant expression induced by icaritin might be a potential therapeutic strategy against OSCC development.

In the present study, we investigated the effect of icaritin on OSCC cell line growth and tried to elucidate its potential molecular mechanisms for the first time. We also used an oral specific carcinogenesis mouse model to observe the ability of icaritin to prevent OSCC development in vivo.

## 2. Results

### 2.1. Icaritin Inhibited Proliferation of OSCC Cells

**Figure 2:**
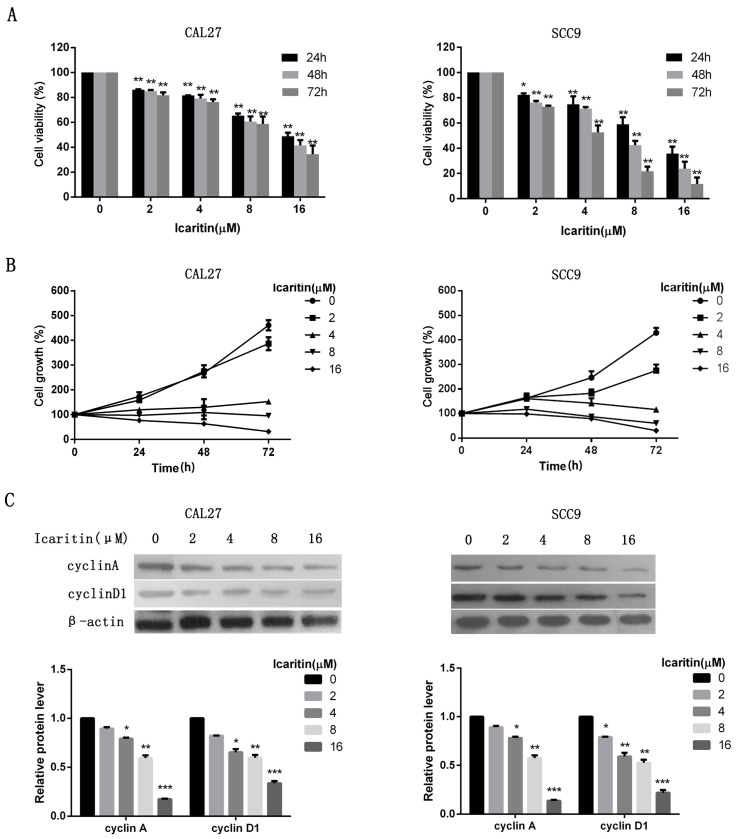
Icaritin inhibited OSCC cells proliferation. (**A**,**B**) Dose and time-response curves showing icaritin effects on OSCC cell lines growth inhibition. CAL27 and SCC9 cells were treated with icaritin at increasing concentrations (2, 4, 8, and 16 μM) for 24, 48, and 72 h. Cell viability was evaluated by CCK-8 assay. Data were expressed as mean ± standard deviation (SD) (*n* = 3, in triplicate). * *p* < 0.05; and ** *p* < 0.01 versus 0 μM group; (**C**) Icaritin downregulated cyclin A and cyclin D1 expression, as evaluated by western blot. β-actin was used as a loading control. Relative density data of each protein was quantified by β-actin using Image J, and the data represented the mean of three independent experiments. * *p* < 0.05; ** *p* < 0.01; and *** *p* < 0.001 versus 0 μM group.

To explore the anti-tumor effects of icaritin on OSCC cells, dose and time -response studies were performed in CAL27 and SCC9 cells. The results showed that icaritin significantly inhibited cell proliferation compared with the control group (*p* < 0.05) (Figure 2A). In particular, icaritin treatment at the concentrations of 8 and 16 μM resulted in a more efficient cell growth inhibition at 48 and 72 h, compared with the inhibition at 24 h (Figure 2B). Icaritin IC_50_ value on CAL27 or SCC9 cells were 15.99 or 10.36 μM (24 h), 11.87 or 6.11 μM (48 h), and 9.93 or 3.95 μM (72 h), respectively. Western blot results showed that icaritin treatment on CAL27 and SCC9 cells reduced the expression of both the cell-cycle progression proteins cyclin A2 and cyclin D1 in a dose-dependent manner (Figure 2C).

### 2.2. Icaritin Induced Apoptosis of OSCC Cells

**Figure 3:**
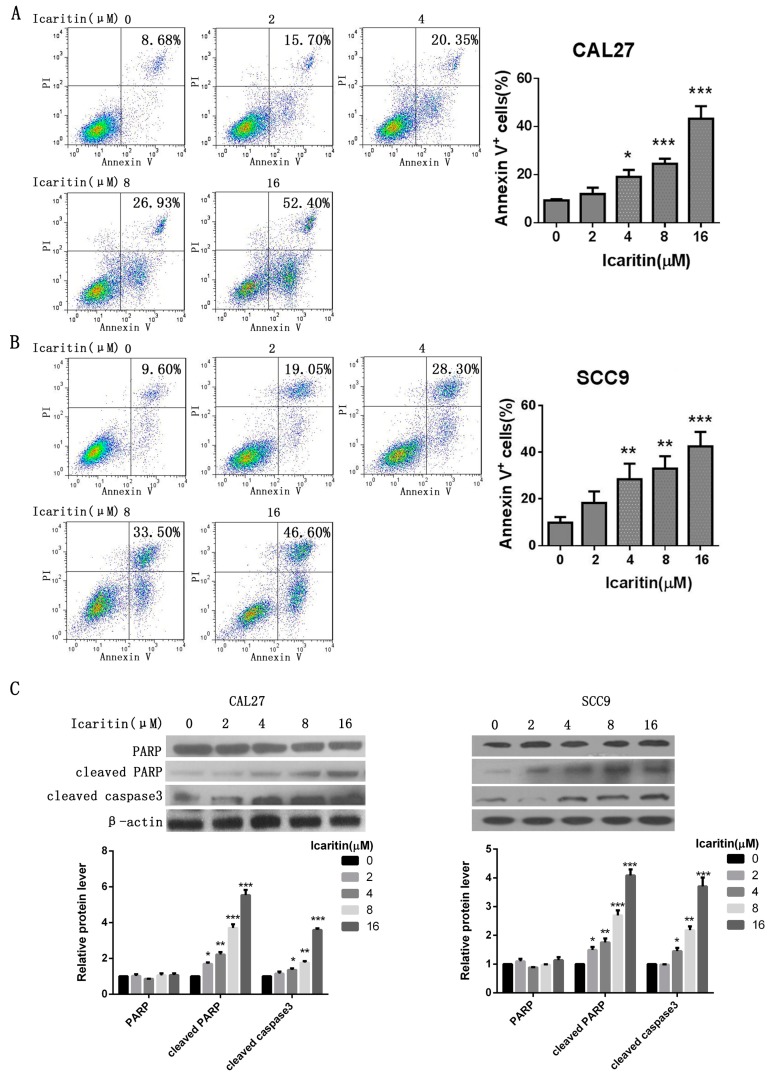
Icaritin induced apoptosis in OSCC cells. (**A**,**B**) OSCC cell apoptosis after icaritin treatment. CAL27 and SCC9 cells were treated with icaritin at indicated doses for 48 h, then were harvested and stained with Annexin-V FITC and PI. Annexin-V positive cells were measured by flow cytometry. Columns represent the average percent of Annexin V positive cells. Data were expressed as mean ± SD (*n* = 3, in triplicate). * *p* < 0.05; ** *p* < 0.01, *** *p* < 0.001. Representative images were shown in the left panel; (**C**) Effects of icaritin on apoptosis related proteins, total and cleaved PARP, and cleaved caspase 3. β-actin was used as a loading control. Relative density data of each protein was quantified by β-actin using Image J, and the data represented the mean of three independent experiments. * *p* < 0.05; ** *p* < 0.01; and *** *p* < 0.001 versus 0 μM group.

Cell apoptosis was remarkably increased in both icaritin-treated CAL27 and SCC9 cells. Annexin-V positive cells were significantly increased in the icaritin treated groups (4, 8, and 16 μM) compared with control group (*p* < 0.05) (Figure 3A,B). Icaritin increased the expression of both apoptosis-related cleaved caspase 3 and cleaved poly-(ADP-ribose) polymerase (PARP) in a dose-dependent manner (Figure 3C).

### 2.3. Icaritin Triggered Autophagy in OSCC Cells

**Figure 4:**
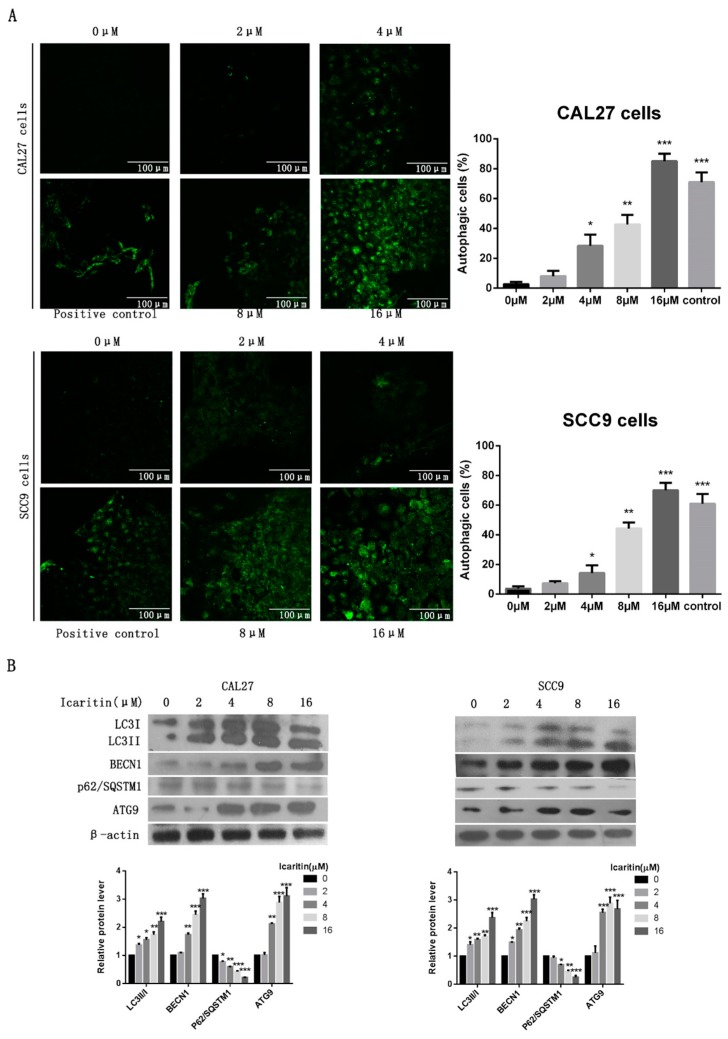
Icaritin triggered OSCC cell autophagy. (**A**) Representative images of MDC staining of CAL27 and SCC9 cells after icaritin treatment at the indicated concentration for 48 h. Punctated fluorescence in the cytoplasm indicated the formation of autophagic vacuoles. Magnification: ×400 for MDC staining. * *p* < 0.05; ** *p* < 0.01; and *** *p* < 0.001 versus 0 μM group; (**B**) CAL27 and SCC9cells were treated with icaritin at different concentration for 48 h, and then LC3I/II, BECN1, p62/SQSTM1, and ATG9 were detected by western blot analysis. β-actin was used as a loading control. Relative density data of each protein was quantified by β-actin using Image J, and the data represented the mean of three independent experiments. * *p* < 0.05; ** *p* < 0.01; and *** *p* < 0.001 versus 0 μM group.

As shown in Figure 4A, punctated fluorescence indicating autophagic vacuoles was significantly increased in a dose-dependent manner in the cytoplasm of icaritin treated OSCC cells. Consistently, western blot results demonstrated that icaritin could induce the conversion of LC3I to LC3II, indicative of autophagic activity, in CAL27 and SCC9cells, increase BECN1 expression, and reduce p62/SQSTM1 expression, both of them autophagy-associate protein (Figure 4B).

### 2.4. Icaritin Inhibited JAK2/STAT3 Signaling in OSCC Cells

**Figure 5:**
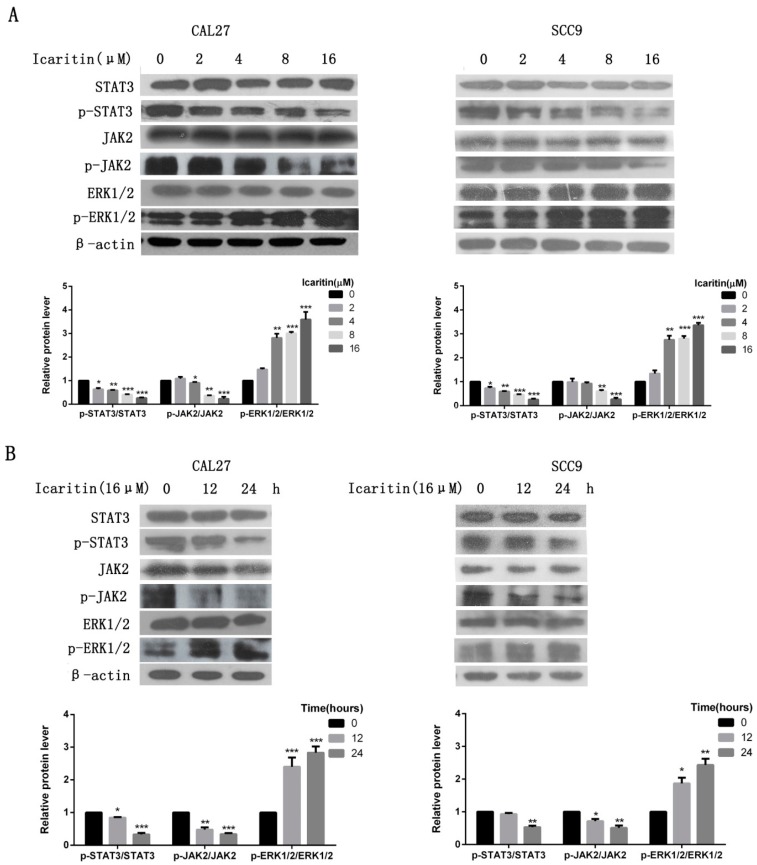
Effects of icaritin on oncogenic signaling pathways in OSCC cells. (**A**) CAL27 and SCC9 cells were treated with icaritin at the indicated doses for 24 h. Protein levels were detected by western blot using the correspondent antibodies. β-actin was used as a loading control. Relative density data of each protein was quantified by β-actin using Image J, and the data represented the mean of three independent experiments. * *p* < 0.05; ** *p* < 0.01; and *** *p* < 0.001 versus 0 μM group. Icaritin inhibited the expression of p-JAK2 and p-STAT3, while p-ERK1/2 expression was upregulated; (**B**) CAL27 and SCC9 cells were treated with icaritin at the indicated interval times. p-JAK2 and p-STAT3 inhibition was increased with time lasting. * *p* < 0.05; ** *p* < 0.01; and *** *p* < 0.001 versus 0 h group.

To elucidate icaritin anti-tumor mechanisms on OSCC cells, several major oncogenic signaling pathways—such as JAK2, STAT3, and ERK—were examined. The results demonstrated that icaritin significantly inhibited the expression of phospho-STAT3 (p-STAT3) in a dose- and time-dependent manner, although it had no effects on total STAT3 protein levels (Figure 5A,B). Phospho-JAK2 (p-JAK2) was also inhibited by icaritin (Figure 5A,B). Besides, p-ERK1/2 level was increased in CAL27 cells (Figure 5A).

### 2.5. Icaritin-Induced Proliferation Inhibition and Apoptosis Were Regulated by STAT3 Signaling in OSCC Cells

**Figure 6:**
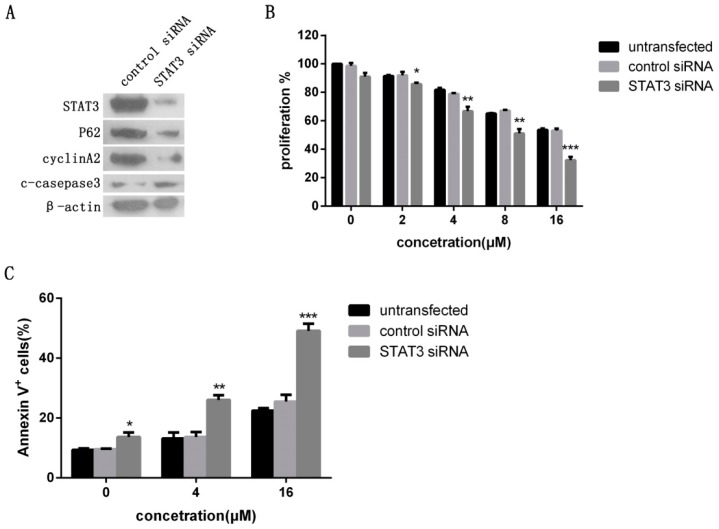
Antitumor effect of icaritin was affected by the levels of STAT3 activity in OSCC cells. (**A**) CAL27 cells were transfected with STAT3 and non-specific siRNA, and the successful transfection was confirmed by western blotting. STAT3 knockdown affected the expression of proliferation and apoptotic proteins; (**B**,**C**) STAT3 silencing enhanced the effects of icaritin on CAL27 cell growth inhibition and apoptosis promotion. CAL27 cells were transfected with either non-specific siRNA or STAT3 siRNA, and then treated with icaritin at the indicated doses for 48 h. Cell proliferation and apoptosis were detected. Data were expressed as mean ± SD (*n* = 3, in triplicate). * *p* < 0.05; ** *p* < 0.01; and *** *p* < 0.001 versus 0 μM group.

To further investigate whether STAT3 signaling activity directly affects the biological effects of icaritin in OSCC cells, CAL27 cells were transfected with siRNA against STAT3 or a control vector (nonspecific siRNA), and the successful transfecttion was confirmed by western blot analysis (Figure 6A). Cyclin A2 and p62/SQSTM1 expressions were clearly decreased after transfection, while cleaved caspase 3 expression increased (Figure 6A). The effect of icaritin in the inhibition of CAL27 cells proliferation was significantly enhanced (*p* < 0.05) (Figure 6B), and icaritin-induced apoptosis in STAT3 siRNA group was significantly increased compared with apoptosis in the nonspecific siRNA group (*p* < 0.01) (Figure 6C). These results indicated that icaritin inhibited cell growth and induced apoptosis of OSCC cell lines in vitro through STAT3 signaling.

### 2.6. Icaritin Inhibited Oral Tumorigenesis Induced by 4NQO and Potential Premalignant Lesions Progression In Vivo

**Figure 7:**
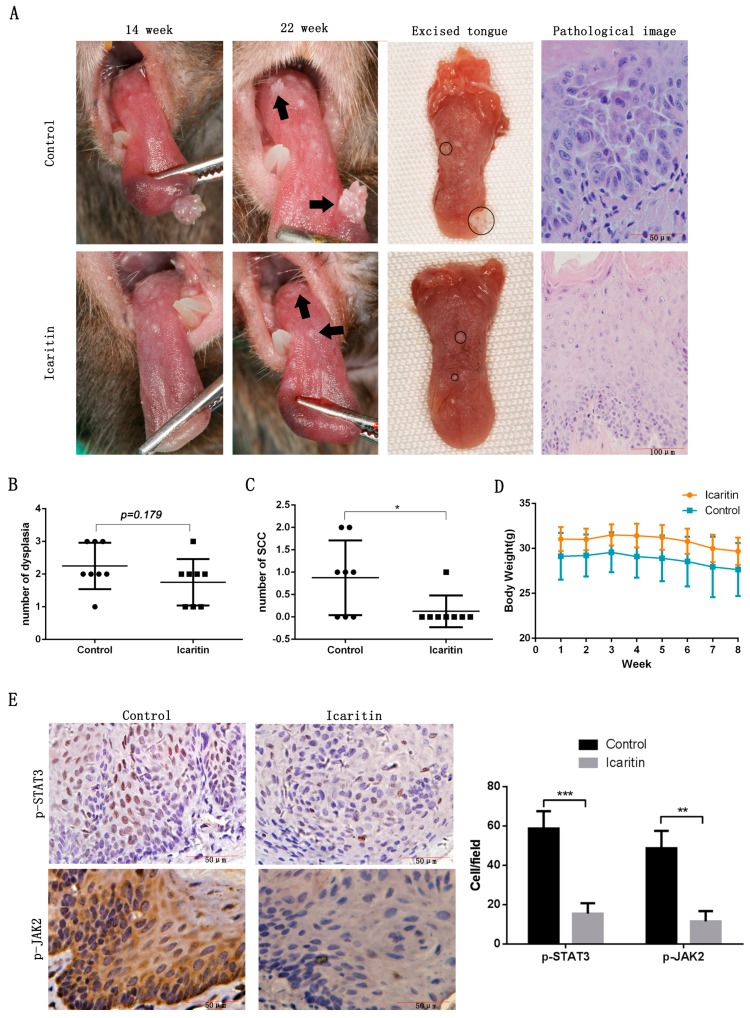
Icaritin decreased the number of oral tumors and prevented the malignant conversion of premalignant lesions. (**A**) At week 22, the lesions in the control group showed a significantly large size compared with the location at week 14 (black arrow). Under higher magnification, the lesions revealed atypical epithelial cells with squamous differentiation and basement membrane partially disappeared. In the icaritin-treated group, the lesion size became smaller (black arrow). Under higher magnification, the epithelium was clearly hyperplastic, while the cells displayed no evident atypia. The lesions on the excised tongue were marked with black circle; (**B**) The total number of lesions (benign and malignant tumors) in the icaritin treated group was less than that in control group, although no significant difference was observed; (**C**) The number of malignant tumors was significantly less in the icaritin-treated mice compared to the untreated group. * *p* < 0.05 versus control group; (**D**) Mice body weight was measured every week during the experiments. Data were expressed as mean ± SD; (**E**) Immunohistochemical analysis of p-STAT3 and p-JAK2 (×400 of magnification). Icaritin significantly inhibited the expression of p-STAT3 and p-JAK2 in the epithelium of the tumor. ** *p* < 0.01; and *** *p* < 0.001 versus control group.

Next, icaritin effect on OSCC progression in vivo was assessed. As show in Figure 7A, the size of the lesions became smaller after a period of eight weeks of treatment with icaritin compared to the beginning of the treatment. In contrast, at week 22, the lesions in the control group showed a large size compared with the location at week 14. Microscopically, most tumors in the icaritin untreated mice developed atypical squamous epithelial cells. The number of malignant tumors in the icaritin-treated group was significantly less than the control group (*p* < 0.05) (Figure 7C). Icaritin also reduced the total number of tumor lesions, although this difference was not statistically significant (Figure 7B). Besides, icaritin treatment did not affect the weight of the mice. At the end of experiment (the 22nd week), the mean body weight was 30.49 g ± 1.40 g in icaritin-treated groups, which was not significantly different from the weight at the beginning of the treatment (the 14th week, 31.03 g ± 1.33 g, *p* > 0.05) (Figure 7D). Furthermore, icaritin treatment clearly reduced the expression of p-STAT3 and p-JAK2 in the epithelium of the tumor compared with untreated control (Figure 7E).

## 3. Discussion

Recently, a new approach in cancer chemoprevention is the exploration of the important pharmacological action of many natural products such as lycopene, curcumin, epigallocatechin-3-gallate (EGCG) and resveratrol, as well as icaritin [14]. Previous studies demonstrated that cell-cycle progression is dynamically and strictly regulated by cyclins, which are critical for normal cell cycle progression [10,28]. Tong et al. indicated that icaritin-induced cell growth inhibition was due to the decrease of cyclin D1 in human endometrial cancer cells [10]. Zhu et al. reported that icaritin reduced cyclin A, cyclin B, and CDK2 expression in multiple myeloma [14]. Our study showed that icaritin clearly reduced cyclin D1 and cyclin A2 expression in a dose-dependent manner. These results suggested that icaritin could regulate cell-cycle related effectors. Tong et al. also observed that icaritin induced apoptosis of Hec1A cells, but the proportion of necrotic cells was not significantly increased, which was consistent with our results [10]. Thus, this result could indicate that growth inhibition exerted by icaritin might be due to apoptosis induction. Apoptosis, a form of regulated cell death, is promoted by the activation of cysteine proteases of the caspase 3 [29]. PARP play a role in DNA repair, while cleaved PARP is considered as a marker of apoptosis and could be induced by caspase 3 [30]. In the present study, we found that icaritin treatment resulted in a significant increment of cleaved caspase-3 and PARP in a dose-dependent manner. Based on the above, these results suggested that icaritin-induced OSCC cell apoptosis was due, at least in part, to the caspase pathway activation.

STAT3 plays a key role in various biological processes, including tumor cell proliferation promotion, survival, tumor invasion, angiogenesis, autophagy, and immunosuppression [21,22,31]. Some studies have indicated the important role of STAT3 in the progression of OSCC [18,32,33]. TGF-α/EGFR autocrine or paracrine activation is frequently observed in OSCC, and STAT3 is a vital signaling network downstream of TGF-α/EGFR [34]. In primary tumors of OSCC patients, STAT3 is constitutively activated and induces expression of cyclin D1, which is associated with cancer cell growth [21]. STAT3 activation also leads to increased cell proliferation, angiogenesis, and multidrug-resistance, as well as reduced cell apoptosis, thus promoting tumorigenesis in OSCC [22,35,36]. In the present study, icaritin clearly inhibited STAT3 activity in OSCC cell lines, associated with upstream p-JAK2 inhibition in a dose- and time-dependent manner. Furthermore, icaritin suppressed the expression of p-JAK2 and p-STAT3 in mice carcinoma tissue, which was consistent with our results in vitro. In addition, STAT3 knockdown enhanced the effects of icaritin on CAL27 cell growth arrest. Finally, cyclin D1, a downstream target of STAT3-regulated genes [18], was significantly reduced in STAT3 knockdown CAL27 cells. In contrast, knockdown of STAT3 in CAL27 cells significantly increased the expression of cleaved-caspase 3. Consistently with these results, Zhu et al. found that silencing of STAT3 not only resulted in a remarkable cell proliferation decreases, but also induced increased apoptosis in icaritin-treated multiple myeloma cells compared with cells treated with nonspecific siRNA [14]. These results suggested that the anti-tumor effects of icaritin were mainly through STAT3 signaling inhibition in OSCC.

Previous studies demonstrated that icaritin could also suppress cancers through other signaling pathways, such as ERK, cell cycle-regulated, and apoptosis signals [10,11,15,16,37]. Previous reports show that icaritin induced ERK1/2 sustained phosphorylation and activation, thus inhibiting the growth of endometrial and breast cancer cells [10,11]. In the present study, icaritin also increased p-ERK1/2 expression in CAL27 cells. In addition, icaritin could induce cell growth inhibition of prostate and lung cancer cells through cell cycle arrest or apoptosis signaling [9,13]. Our initial results showed that icaritin treatment reduced cyclin A2 and cyclin D1, while it activated cleaved-PARP and cleaved-caspase 3. Therefore, besides JAK2/STAT3 signaling, ERK, cell cycle and apoptosis signal pathways might be also involved with icaritin treatment in OSCC. 

Many stress pathways elicit autophagy and apoptosis simultaneously within the same cell. Autophagy induction is exacerbated when apoptosis is suppressed [38]. Some studies have reported that autophagy and apoptosis could be sequentially induced by anti-tumor drugs in cancer cells [39,40]. In the present study, the results indicated that icaritin induced autophagy in a dose-dependent manner. As far as we know, this is the first report showing the effects of icaritin on autophagy in OSCC cells. As distinct self-destructive processes, autophagy and apoptosis have a very complex interaction. Although, in some cases, autophagy may help to induce apoptosis or necrosis, and could even promote cell death itself. Most studies support the survival role of autophagy in chemotherapy-induced cell death, suppressing apoptosis [41,42]. Therefore, the interference of the interplay between autophagy and apoptosis has an important role in drug treatment of cancer. Icaritin could induce autophagy and apoptosis via STAT3 signaling inhibition in OSCC. Some studies demonstrated that suppressing autophagy can enhance the efficacy of blocking STAT3 signaling on the apoptosis of OSCC cells [20,40]. Thus, we speculated that combining icaritin with autophagy inhibitor may improve the therapeutic efficiency for cancer, which is worth further exploration in the future.

Oral cancers are often associated with stepwise accumulation of genetic and epigenetic alterations, generally triggered by exposure to chronic tobacco use or betel nut chewing [34]. During this multistep carcinogenesis, premalignant lesions are associated with a likely progression to cancer. [6]. As the rate of malignant conversion of oral leukoplakia is 0.13%–34%, it would be suitable for studying chemopreventive agents by targeting these lesions [5,43]. Previous studies showed that oral administration of 4-NQO could induce the formation of dysplastic lesions and OSCC in mice [33,44]. In addition, the histological and molecular changes during OSCC carcinogenesis in this model were similar to human OSCC [33]. In the present study, we used this model to mimic the process of oral carcinogenesis in humans. Our results showed that the number of malignant tumors in the treated group was significantly less compared with the control group. Thus, icaritin could suppress the progression of oral epithelial dysplasias into carcinomas. Grandis et al. found that the incidence of total preneoplastic and neoplastic lesions significantly decreased due to erlotinib treatment at the beginning of 4NQO exposure [33]. In the present study, the total number of dysplasia also decreased after icaritin treatment, although a statistical difference was not observed between icaritin-treated group and control group, which might be due to the short icaritin time of exposure. This evidence suggested that icaritin efficacy could be improved through its long-term use. More importantly, as a natural product, icaritin showed few adverse reactions such as body weight loss in vivo. Taken together, icaritin may become an attractive chemopreventive agent to hamper the progression of premalignant lesions converting into OSCC.

## 4. Materials and Methods

### 4.1. Proliferation Assay

Human OSCC cell lines CAL27 and SCC9 were cultured in DMEM or DMEM/F12 (Gibco, Carlsbad, CA, USA) respectively, with 10% fetal bovine serum (FBS; Gibco). Cells were incubated in a humidified atmosphere of 95% air and 5% CO_2_ at 37 °C. Cells were seeded at a density of 3 × 10^3^ cells per well in 96-well culture plates. After overnight incubation, the cells were treated with either DMSO (vehicle control; Sigma, St. Louis, MO, USA) or icaritin at different concentrations (>98% pure; Baoji Herbest Bio-Tech Corporation, Baoji, China) (0, 2, 4, 8, and 16 μM) for 24, 48, and 72 h. Cell proliferation was then detected using cell counting kit-8 (CCK-8; Dojin Laboratory, Kumamoto, Japan) according to the manufacturer’s instructions. Absorbance was measured at 450 nm using a 96-well multiscanner autoreader (Thermo Fisher Scientific, Waltham, MA, USA). Then, the inhibition efficiency of icaritin and the OSCC cells growth curves were calculated with the same optical density (OD) values in different ways.

### 4.2. Apoptosis Assay

CAL27 cells and SCC9 cells (3 × 10^5^) were seeded in 6-well plate. Next, cells were incubated with icaritin for 48 h at the concentrations previously mentioned. Floating and attached cells were collected and cell apoptosis was assessed using Annexin V-FITC and PI apoptosis detection kit (BD Biosciences, San Diego, CA, USA) by FACSCaliber flow cytometry (Becton-Dickinson, Franklin Lakes, NJ, USA), according to the manufacturer’s instructions. Data were analyzed by FlowJo software (Treestar, Ashland, OR, USA).

### 4.3. Autophagy Assay

The increased acidic vesicular organelles (AVOs) are correlated with increased autophagosomes, which could indicate the formation of autophagolysosomes. Monodansylcadaverine (MDC) (Sigma, St. Louis, MO, USA) staining was employed to detect the AVO formation in the autophagic process. CAL27 cells and SCC9 cells were seeded on slides in 24-well plate and incubated with icaritin for 48 h at the concentrations previously mentioned. CAL27 cell and SCC9 cell autophagy was induced by 1 μM rapamycin for 3 h to obtain the positive control. Autophagic vacuoles of treated cells were stained with 0.05 mM MDC and incubated at 37 °C for 30 min. After incubation, cells were washed by phosphate buffered saline (PBS) three times, and observed by a laser scanning confocal fluorescence microscopy (Olympus, Tokyo, Japan).

### 4.4. Western Blot

CAL27 cells and SCC9 cells were treated with icaritin for 48 h at the concentrations previously mentioned, then were harvested, washed immediately with ice-cold PBS, and lysed by RIPA buffer containing PMSF (Beyotime Biotechnology, Shanghai, China) and phosphatase inhibitors (Beyotime Biotechnology). The whole cell lysates were collected after centrifugation at 12,000× *g* for 15 min at 4 °C, and protein concentration in the supernatants was determined by BCA assay kit (Beyotime Biotechnology). Equal amounts of protein (6 μg/lane) were loaded onto 12% SDS-polyacrylamide gels, subjected to electrophoresis, and transferred to PVDF membranes (Millipore, Billerica, MA, USA). After blocking with 5% nonfat milk for 1 h, membranes were incubated overnight at 4 °C with primary antibodies against proliferation related proteins cyclin D1 and cyclin A2 (Cell Signaling Technology, Danvers, MA, USA); apoptosis related proteins cleaved-caspase 3 and cleaved-PAPR (Cell Signaling Technology); autophagy related proteins p62/SQSTM1, BECN1, LC3B, and ATG9B (Abcam, Cambridge, MA, USA) and STAT3 associated proteins STAT3, phospho-STAT3, phospho-JAK2, phospho-extracellular signal-regulated kinase 1/2 (p-ERK1/2) (Cell Signaling Technology). After washed three times with TBST, membranes were then incubated with HRP-labelled secondary antibody for 1 h at room temperature. The protein bands were visualized using ECL reagent. β-actin was used as the internal loading control. Western blot experiments were repeated at least three times.

### 4.5. siRNA Interference for STAT3

CAL27 cells were seeded onto a culture plate and transiently transfected with human STAT3 siRNA (5’-CCGTGGAACCATACACAAA-3’) and negative control siRNA according to the manufacturer’s transfection protocol (RiboBio, Guangzhou, China). After 48 h transfection, cells were treated with the indicated concentration of icaritin for 48 h, and cell viability and apoptosis were measured.

### 4.6. Animal Studies

The animal protocol was approved and supervised by the Ethical Board of the Center for Laboratory Animal Science at the Wuhan University (No. 00133334; 14 April 2015) and conducted according to the Guidelines for the Care and Use of Laboratory Animals. Female C57BL/6 mice (eight weeks old) were housed in a temperature-controlled room (25 °C) under 12 h/12 h light/dark cycle and have access to food and water ad libitum. To explore the OSCC preventing effect of icaritin in vivo, an OSCC mouse model was established. 4-Nitroquinoline-1-oxide (4NQO; Sigma, St. Louis, MO, USA) was added in the drinking water (50 μg/mL). All mice received a full oral examination under anesthesia every week. After 14 weeks, 16 mice with at least one premalignant lesion in the tongue were reverted to regular water, and randomly divided into treatment and control groups, which received intraperitoneal (i.p) injections of icaritin (10 mg/kg) dissolved in 0.3% sodium carboxymethylcellulose (CMC-Na; Sigma, St. Louis, MO, USA) or an equal amount of solvent once every other day. All animals were euthanized at week 22, and tissues were fixed and embedded in paraffin for histopathological analysis and subsequent immunohistochemical staining.

### 4.7. Immunohistochemistry Assay

Concentrations of primary antibodies were used for monoclonal mouse anti-p-STAT3 1:400 and anti-p-JAK2 1:200, which were purchased from Cell Signaling Technology. Paraffin sections were dewaxed and rehydrated through a series of xylene, graded alcohol, and water immersion steps. The deparaffinized and hydrated slides were treated with 3% H_2_O_2_ at 37 °C for 20 min to block endogenous peroxidase activity and then washed with PBS. Slides were treated with 5% normal goat serum for 30 min to block non-specific binding, and incubated with primary antibodies at 4 °C overnight. After washing with PBS, they were incubated with biotinylated goat anti-mouse or goat anti-rabbit IgG antibody (Zhongshan Golden Bridge Ltd., Beijing, China) for 15 min and washed again with PBS. DAB detection kit (Zhongshan Golden Bridge Ltd., Beijing, China) was used for staining. The slices were visualized for 1 min following staining, then re-stained with hematoxylin and sealed. Cells with brown staining were considered positive. The staining intensity and positive ratio of the mentioned proteins were observed under microscope by two independent scorers blinded to the clinical parameters.

### 4.8. Statistical Analysis

Data were expressed as means ± standard deviation (SD) for the indicated number of separate experiments. IC_50_ for icaritin was calculated from linear transformation of dose-response curves. The differences between experimental and control groups were analyzed using Student’s *t* test or one-way ANOVA. All statistical analyses were performed using GraphPad Prism version 5.0 (Graphpad Software Inc., La Jolla, CA, USA) and SPSS 17.0 (Chicago, IL, USA). Statistical significance was defined as *p* < 0.05.

## 5. Conclusions

Our findings indicated that icaritin inhibited proliferation and induced apoptosis and autophagy in OSCC cells mainly through STAT3 signaling inhibition. Remarkably, icaritin could prevent oral premalignant lesion conversion to squamous cell carcinomas in vivo. Hence, the natural product icaritin could be considered as a promising agent in OSCC treatment and prevention.
